# Now We Are Six

**DOI:** 10.1371/journal.pntd.0001862

**Published:** 2012-10-25

**Authors:** Peter J. Hotez, Serap Aksoy

**Affiliations:** 1 Sabin Vaccine Institute and Texas Children's Hospital Center for Vaccine Development, Departments of Pediatrics and Molecular Virology & Microbiology, Baylor College of Medicine, Houston, Texas, United States of America; 2 National School of Tropical Medicine, Baylor College of Medicine, Houston, Texas, United States of America; 3 James A. Baker III Institute for Public Policy, Rice University, Houston, Texas, United States of America; 4 Department of Epidemiology and Public Health, Division of Epidemiology of Microbial Diseases, Yale University School of Public Health, New Haven, Connecticut, United States of America

*When I was One, I had just begun.*

*When I was Two, I was nearly new.*

*When I was Three, I was hardly me.*

*When I was Four, I was not much more.*

*When I was Five, I was just alive.*

*But now I am Six, I'm as clever as clever,*

*So I think I'll be six now for ever and ever.*

From *Now We Are Six* by A. A. Milne [1]

**Figure 1:**
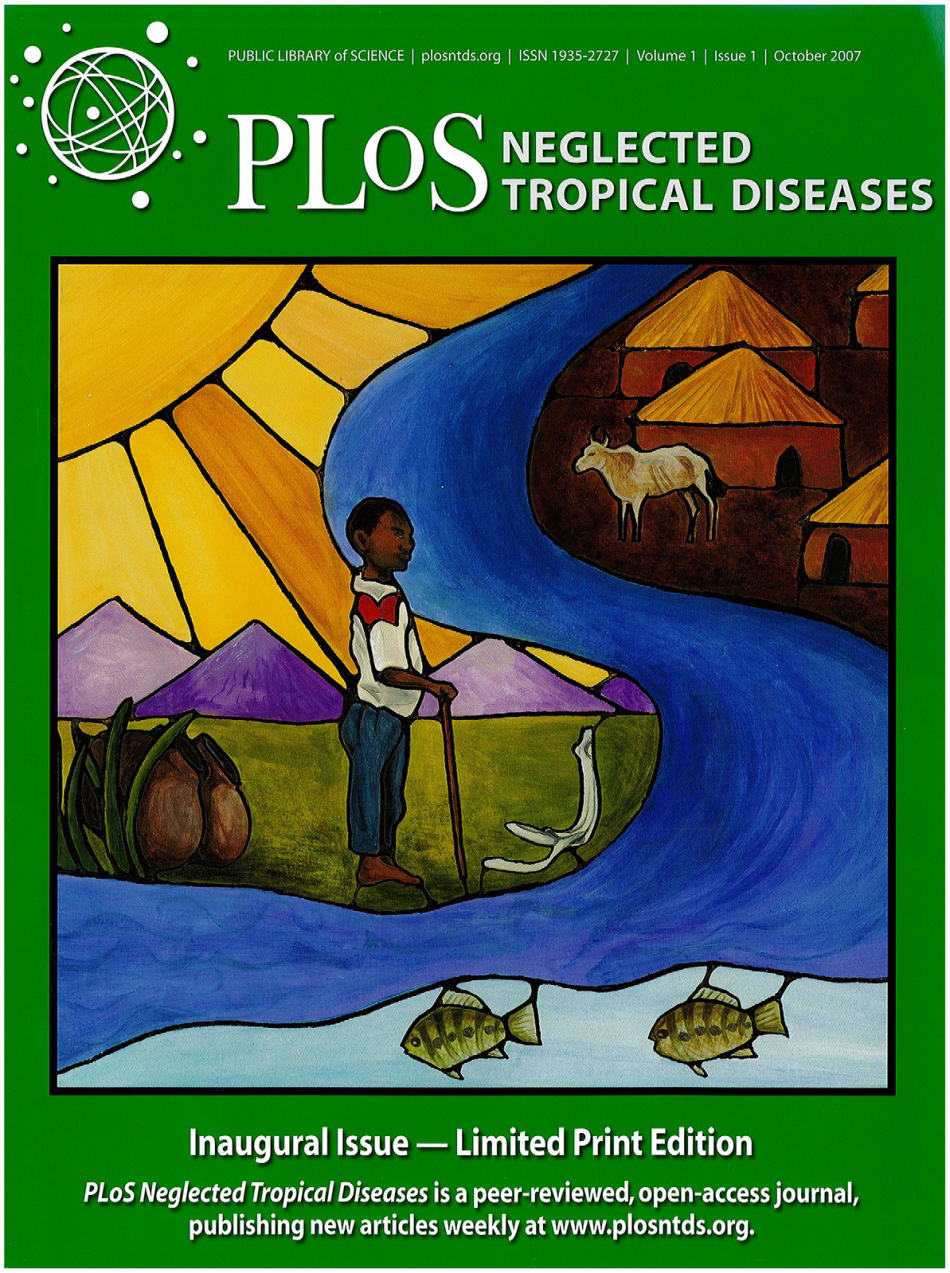
Cover of the *PLOS NTDs* inaugural issue, limited print edition. Art by Ms. Emma Burns, “A Ray of Hope.”

This year we will begin our sixth year of publishing *PLOS Neglected Tropical Diseases*, the first open-access journal for the community of biomedical and social scientists, public health experts, and health care providers committed to studying the neglected tropical diseases (NTDs) (Figure 1). Jumpstarted with seed funds from the Bill & Melinda Gates Foundation, together with an editorial staff and a PLOS board of directors dedicated to editorial and journal capacity building in the world's low- and middle-income countries (LMICs), *PLOS NTDs* began in 2007 with an editorial entitled A New Voice for the Poor [2]. From the outset, *PLOS NTDs* worked to create a unique vision for publishing tropical medicine papers that was different from other journals in the field. By maintaining our dedication to the concept of open access, we hoped that *PLOS NTDs* would be read by a wide community of NTD experts while simultaneously attracting new people to the field.

**Figure 2:**
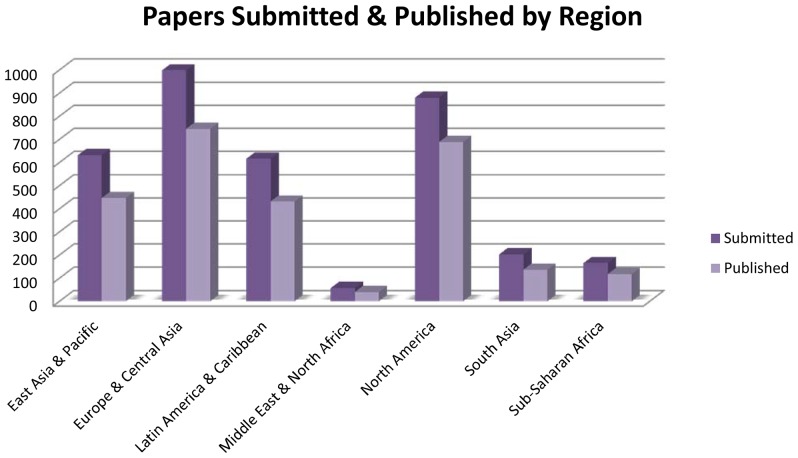
Papers submitted and published by region between October 2007 and July 2012.

We began especially eager to embrace a larger community of scholars from LMICs, and to an extent we have achieved some successes on that front. Today, roughly 30 percent of our editorial board members are from LMICs. Brazil, China, and India are among the seven leading countries from which corresponding authors have submitted papers to *PLOS NTDs* since its founding (Table 1), and Argentina, Colombia, Mexico, and Thailand are among the top 20 countries submitting papers to *PLOS NTDs*. However, our editorial staff and board feel that we could improve our endemic country outreach, and we are aggressively seeking to expand the editorial board representation and papers that come from low-income countries in sub-Saharan Africa, the Middle East and North Africa, and South Asia, where submissions have been far fewer (Figure 2).

**Table 1:** Top countries by number of submissions.

Rank	Country	Number of Submissions
1	United States	655
2	Brazil	364
3	United Kingdom	213
4	France	167
5	India	131
6	China	126
7	Australia	107

We are deeply grateful to the Gates Foundation for their initial support, which ended more than two years ago. Today, we are pleased to report that we are at the “break-even point” financially. Currently, we receive close to 100 submissions each month—more than double the number of papers from when we first started. Unlike some of the other *PLOS* journals, which in some cases only accept one in ten papers submitted, our goal is to be more inclusive so as to strongly embrace authors and papers from LMICs. Today our acceptance rate at *PLOS NTDs* is roughly 50 percent, and whenever possible we try to provide intensive editorial support for submissions by LMIC authors.

We are proud of the fact that our capacity building efforts have not occurred at the expense of quality. For the last three years, since we became eligible to receive a Thomson Reuters citation impact factor rating, our journal has had the highest ranking of any tropical medicine journal. We are pleased to report that this success has also not come at the expense of other tropical medicine journals, whose impact factors have also mostly increased over the years. Shortly after our launch, Dr. Richard Horton, *The Lancet* editor-in-chief, wrote a strong endorsement of our journal and our efforts at global outreach. We were very grateful for that support, and in that spirit we also hope to ensure that our editors and staff “lift all boats” in the area of tropical medicine publishing, especially for those journals and organizations committed to open access. After we began, the open-access journal *Parasites & Vectors* was launched by BioMed Central, and we especially want to wish them success, as well as other journals such as the *American Journal of Tropical Medicine and Hygiene*, which now offers open-access options.

Ultimately, the leadership at PLOS feels strongly that there are more informative metrics available than those offered by conventional impact factor ratings. For instance, along with the other PLOS journals, we have implemented a system of article-specific metrics in order to determine the number of times that our articles are downloaded and viewed. We have determined that 20 of our *PLOS NTDs* articles have been downloaded at least 5,000 times each by our readers, while four articles have been downloaded more than 10,000 times, including articles on the origins of the treponematoses, NTDs in sub-Saharan Africa and Latin America, and even neglected infections of poverty in the United States. Our most widely viewed article has more than 30,000 downloads. We also follow blogosphere coverage and coverage by major media outlets—stories about *PLOS NTDs* appear regularly in leading newspapers such as the *New York Times* and the *Guardian*, as well as electronic media—in addition to community ratings and expert assessments.

A word about journal scope: since our beginning we have tried to stay focused on the major NTDs afflicting the “bottom billion”, i.e., the world's 1.4 billion people who live in poverty and are disproportionately affected by these conditions [2]. We have subsequently responded to an increasing demand from the arbovirus community (especially regarding dengue papers) and experts in neglected bacterial and fungal infections (e.g., leptospirosis, mycobacterial infections such as Buruli ulcer and leprosy, and paracoccidioidomycosis) by increasing our editorial board representation in these areas. We also look forward to continuing our long-standing commitment to papers on neglected helminth and protozoan infections. In contrast, we have largely avoided papers that deal with falciparum malaria unless they also mention collateral effects on the NTDs. Instead, we feel there are excellent journals already out there that can handle such papers, including the open-access *Malaria Journal*. However, we have listened to the scientists and health professionals who make a compelling case that vivax malaria is a true NTD and have been reviewing papers on this disease for several years now. We anticipate that the scope of *PLOS NTDs* will continue to evolve and invite our readers to visit the journal scope section of our website and even to submit editorials that make a strong argument why the scope may require amendment or adjustment.

As our journal expands and grows, we are working hard to ensure that papers submitted to *PLOS NTDs* receive a timely review and decision. Together with our deputy editors and associate editors, the PLOS editorial staff based in San Francisco and now also Cambridge, United Kingdom, meets continually to review its procedures and seeks ways to improve the quality of our reviews and shorten timelines. Currently, new research articles receive their first decision on average within 40 days of initial submission. Manuscripts that do not get sent to review receive a rapid response form the journal; on average, these decisions are sent to authors within 13 days of submission. Once a paper has been accepted, it spends an average of 46 days in production until publication. However, we feel these numbers can also be improved, and in the coming months we look forward to implementing revised or new practices and procedures to achieve those goals.

In addition to its long-standing commitment to excellent and high quality science, in the coming years *PLOS NTDs* will continue a strong emphasis on advocacy and shaping NTD health and economic policy. We feel that our papers have helped to inform, advocate, and ultimately shape important financial commitments from the US and UK governments to support global NTD control and elimination efforts, as well as research and development for new control tools, i.e., drugs, insecticides, diagnostics, and vaccines. We believe that *PLOS NTDs* papers have also positively influenced government leaders and key private donors.

In this issue, we present two special collections of articles published to date that have received a disproportionately frequent number of viewings. The first is a series of review articles contributed by Dr. Hotez and his colleagues on geopolitical aspects of the NTDs, including information on the distribution of the most important NTDs in South Asia, Central Asia, Oceania, Latin America, sub-Saharan Africa, the Middle East, the US, and the Canadian Arctic, published in *PLOS NTDs* over the last five years. The second collection, “Top Ten”, celebrates the success of the most viewed research articles—the top two in each year of our existence to date. We are also looking to feature and celebrate the achievements of our global community of NTD scientists and institutions with a series of Historical Profiles and Perspectives that will be published in the course of the coming year. The inaugural Historical Profile will highlight Centro Internacional de Entrenamiento e Investigaciones Médicas (CIDEIM) from Cali, Colombia. We want to get to know you better and learn from your experiences and achievements–please contribute to this series!

Finally, we want to thank our outstanding managing editorial staff led by Marina Kukso in the San Francisco office, and the unwavering support of the PLOS leadership and board. To restate, our major purpose remains to serve the NTD community of scholars and public health experts. We exist for your benefit and look forward to hearing from you on the ways in which we can improve how we can help the world's “bottom billion” afflicted by the NTDs.
